# Navigating radiography as a larger-bodied patient: a qualitative exploration

**DOI:** 10.3389/fpubh.2026.1803012

**Published:** 2026-06-15

**Authors:** Fay Manning, Christine Heales, Poppy Ulett, Amy Hancock

**Affiliations:** 1 Department of Health and Care Professions, Faculty of Health and Life Sciences, University of Exeter Medical School, Exeter, United Kingdom; 2 School of Nursing and Midwifery, Faculty of Health, University of Plymouth, Plymouth, United Kingdom

**Keywords:** healthcare avoidance, larger bodies, radiography, radiotherapy, weight stigma

## Abstract

**Introduction:**

Radiographic technologies pose barriers to the care of patients with larger bodies both in medical imaging and radiotherapy. Whilst research exists on staff attitudes, there is a lack of understanding of patient experiences. Therefore, this study investigated the qualitative experiences of larger bodied adults who have attended radiographic services within the United Kingdom (UK).

**Methods:**

A cross-sectional survey was distributed via social media. The anonymous survey collected demographic data and invited participants to describe their most impactful experience within radiographic services. Thematic analysis was conducted with three researchers agreeing the final themes.

**Results:**

Ninety-two participants completed the survey. Most respondents (98.9%) reported their experience with medical imaging, while 16.3% reported their radiotherapy experience. Responses were categorised into three time periods (pre-appointment, during and ongoing) as well as ‘societal beliefs’ and ‘suggestions of improvements’. Example themes included ‘sense of self’, ‘exacerbating factors’ and ‘emotional impacts’. The experiences of both taller and larger individuals highlighted recurrent issues in access and care received, as well as some examples of good practice. The outcomes of such experiences led to health care avoidance in some cases, with others either fearful or hopeful of their future health interactions depending on how they were treated.

**Discussion:**

This study highlights clear shortcomings in the care for patients with larger and taller bodies in radiographic services. Issues ranged from stigmatised staff attitudes to unsuitable equipment, often leaving patients feeling excluded or blamed. Impacts of poor practice and healthcare avoidance are widespread, and therefore these issues need immediate attention.

## 1 Introduction

The design of healthcare systems, specifically in publicly-funded healthcare systems, aims to serve the population that exists within (1, 2). Where discrepancies or limitations exist, healthcare provision can perpetuate or create inequalities. Within the National Health Service (NHS) in the United Kingdom (UK) there is an aim to remove inequalities in health by prioritising equitable access and experience with optimal outcomes for all. Part of this involves the identification of areas where healthcare systems are contributing to these inequalities.

One such limitation is that of technologies within radiography. Whether in terms of bed weight limits, Magnetic Resonance Imaging (MRI) bore sizes or associated ionising radiation dose, patients with larger bodies face inequalities in their access to care (3–5). Larger individuals are likely to already be experiencing inequalities in care due to weight/size stigma and fatphobia (6, 7), making it harder for them to access diagnostic tests and treatment (8, 9).

The biases that health care professionals hold across a range of disciplines are quite widely reported. Stereotypes of those of larger size being ‘stupid’, less competent, ‘lazy’ and lacking self-discipline (6, 10) accompany the belief that often those who are larger are to blame for their size and any poor health presentations (11). These views have also been identified within the radiographic workforce. Australian radiographers have been found to blame larger patients for poor image quality, found larger patients more challenging and felt less capable of providing adaptations (12). Similarly, UK student radiographers reported low confidence working with larger patients, finding it ‘frustrating’ (13).

Less examined in the literature, are larger bodied patients’ experiences of radiography (medical imaging and radiotherapy). The first paper of this study – known as Larger Bodies in Radiography (LBinRAD) – was one of the first published to examine patient perspectives; this however focused on quantitative data primarily relating to equipment and utility (14). Another recent a study from Norway focused specifically on ‘fat’ individuals’ experiences of MRI, utilising semi-structured interviews of nine individuals (15). This study highlights four key themes of (1) My body size: a material obstacle outside the margins, (2) To endure the MRI: a historical repeat of distress, (3) Dealing with discomfort and (4) Being the invisible burden. Whilst these papers begin to paint the story of the larger bodied patients’ experience, a wider understanding of cross modality (including radiotherapy) experience is still lacking. This study therefore aimed to investigate the qualitative personal experiences as reported by individuals living within larger bodies who have undergone radiographic services (medical imaging and radiotherapy) as an adult within the UK.

### 1.1 Note on language

Language relating to body size is contested, with different terms carrying distinct social, cultural, and political meanings (16). In this study, the term *“larger bodies”* is used in line with discussions with our Patient and Public Involvement and Engagement (PPIE) contributors, who identified this phrasing as relatively neutral. Specifically, it was considered to avoid medicalised framing while not assuming alignment with fat-positive or activist positions.

PPIE contributors did not express a strong preference regarding person-first versus identity-first language. Given the lack of consensus across communities and within the literature, person-first and identity-first constructions are used interchangeably in this paper.

The term “ob*sity” is used only once, in reference to perspectives that explicitly adopt a medicalised model. The term “ob*se” is also used once in reporting results from a paper which used this wording. Additionally, the term *“higher weight”* appears in limited instances where it reflects the terminology used in cited studies.

Consistent with qualitative approaches, these choices reflect an awareness that language is not neutral and that meanings may shift across contexts. Terminology has therefore been selected to balance clarity, sensitivity, and fidelity to both participant perspectives and the existing literature.

## 2 Methods

Institutional ethical approval was achieved (reference 1,870,378). All participants provided informed consent prior to completion of the survey. Consent was provided by participants using a tick box prior to entry into the study. It followed the provision of a participant information form and researchers’ contact details and included permission for the publication of anonymized quotes.

### 2.1 Survey design

A cross-sectional survey utilising closed and open-ended questions was designed and prepared using Microsoft forms. The survey was piloted with our PPIE representatives and peer researchers. Piloting (two female and two non-binary individual who identified as larger bodied) helped clarify the wording and readability of questions to ensure understanding.

Eligibility criteria included 18-years-old and over adults identifying as ‘larger bodied’; co-defined with Patient and Public Involvement and Engagement PPIE representatives as anyone wider, taller, or broader than the ‘average’, not just those who are ‘plus size’. The PPIE groups specifically advocated for the inclusion of ‘taller’ individuals, a group of individuals we had not explicitly considered previously. This definition of ‘larger bodies’ was important to differentiate the concept and experiences of ‘size stigma’ from weight, and the understanding that body shape and composition differ across cultures, ethnicities and nationalities.

Participants also were required to have had experience (as an adult) of radiographic (medical imaging or radiotherapy) within the UK, either NHS or private. Sonography and MRI were included within the definition of medical imaging as well as approaches utilising radiation (X-ray, CT, etc.).

The first section of the survey contained questions relating to eligibility criteria and informed consent. Demographic questions and aspects focusing on facilities and equipment have been reported previously (14).

Respondents were asked whether they had experience of medical imaging and/or radiotherapy and were then asked to complete the remainder of the survey based on a single, most impactful experience. Questions were designed to ask as neutrally as possible about equipment and utilities, imaging or treatment technology and accessories, as well as staff interactions. A copy of the questionnaire is provided as Supplementary material 1.

In recognition of the potential distress respondents could experience by recounting healthcare experiences, a range of signposting resources was collated in collaboration with PPIE representatives. A debriefing guide for the researchers was also formulated (both included in Supplementary material 3).

### 2.2 Recruitment

The survey was advertised via social media sites [X (formerly Twitter), Facebook, Instagram and TikTok] using respondent-driven snowballing techniques. Adverts included images, videos and frequently asked questions (FAQ’s) distributed via study accounts, and personal accounts of study team members. PPIE representatives also chose to share the survey with their online communities.

A recruitment target of 100 respondents was set based on the principles of information power (17), recognizing that participants, while experts in their own experiences, would each contribute insight limited to individual encounters. As such, a larger sample was required to capture sufficient breadth and variation, particularly given the inclusion of both medical imaging and radiotherapy contexts. The survey was open for a period of 2 months (10 August 2023–13 October 2023) with the option to extend for a third if target recruitment had not been met.

A donation on behalf of respondents to support a tree planting initiative in the UK was included as an incentive. The choice of charity was decided in collaboration with PPIE representatives.

### 2.3 PPIE

We recruited four PPIE representatives (2x white females, 2x white non-binary individuals) who identified as larger-bodied. Representatives were engaged individually rather than as a group based on their preferences. Contributions included reviewing and providing feedback on study design, ethics, survey questions, participant information, consent form, signposting resources and recruitment literature.

A second, larger group of PPIE representatives were recruited through an online platform (People in Research) to discuss results in two online meetings. Of the 27 representatives, 17 identified as female or assigned female at birth (AFAB) and 2 as male. Self-identified ethnicity also included White, South Asian, Black, Indian, Pakistani and Bangladeshi. Further discussion of PPIE involvement can be found in this publication Hancock et al., 2026 (18).

### 2.4 Analysis

Demographic data is presented descriptively, with simple coding of themes (e.g., sex and /or gender, self-description of body size, ethnicity).

Qualitative analysis was undertaken in line with thematic analysis (19). The analysis was conducted with an experiential (essentialist) framework, aiming to represent participants’ reported experiences while acknowledging the interpretative role of the researchers. The analysis was broadly inductive, guided by the overarching research question (“what are the experiences?”) rather than by prior theory or literature, although it was necessarily shaped by the researchers’ perspectives. Coding was primarily semantic, focused on participants’ explicit accounts, though some interpretative decisions were made (for example, classifying “no impact” responses as neutral rather than positive, reflecting an assumption that equitable care represents the baseline expectation). Experienced qualitative researchers FM and AH led the analysis, with contributions from PU. Researchers familiarised themselves with the free text responses and undertook initial line-by-line indicative coding across the dataset. The researchers met intermittently to discuss codes and group them into themes, resulting in an iterative series of coding and re-coding. Final themes and codes were agreed upon between the researchers, with any discrepancies in coding or interpretation resolved through discussion and consensus. Themes were presented to two PPIE groups, who noted that the findings resonated with their experiences. While not intended as a process of validation (20), this discussion provided additional confidence that the analysis reflected issues of relevance to those with lived experience, serving as a form of sense checking.

### 2.5 Reflexivity

As a study team, we recognise that we are not representative of those we are seeking to understand. As a team of white, straight-sized women, we acknowledge that there is an intersection of race, gender and disability that is influential on the experiences of those living in larger bodies. This recognition is part of the motivation behind recruiting a second PPIE group to discuss results and interpretations with, specifically focusing on increasing representations from diverse peoples.

We also acknowledge that the ‘larger-bodied’ experience is diverse. There are those who are embracing (often referred to as ‘fat positive’ or ‘body neutral’), those who subscribe to the medical model of ‘ob*sity’, and further those who are fat-phobic, even to themselves. We were aware that we wanted to capture experiences from all, without passing judgment on their stance, whilst also protecting respondents from unintended shaming through the types of questions asked.

Lastly, all authors have worked clinically and three are also lecturers (FM, AH and CH), which may have shaped our expectations of baseline standards of care. This professional perspective likely influenced interpretative decisions, for example classifying “no impact” responses as neutral rather than positive, whereas individuals with prior negative healthcare experiences might view such responses more positively.

## 3 Results

A total of 101 responses were gained, 92 of which were complete. Full demographic information and terminology of self-described characteristics are reported in Heales et al. (14). Respondent ages ranged from 18 to 85 years and the majority of respondents identified as female (79.3%) and white (94.6%).

The majority of respondents identified as being larger in some way when asked about their self-identified body shape (92.4%), and 42.4% described being above average height (>5.7 ft. for women and non-binary individuals and >6.0 ft. for men). For inclusivity reasons, the question on body shape did not ask for specific measurements; however, 15.2% chose to give a weight or clothing size, and a further 17.4% used a description and measurement.

It should be noted that all those who identified as having a smaller or lean body shape categorised themselves as taller, thus making them eligible for this study.

To support the understanding of the subpopulation of those above average height in our analysis and discussion we use the following terms under the umbrella of ‘larger bodies’:

- Height-defined
- Non-height-defined

### 3.1 Qualitative results

Qualitative themes were grouped into three primary temporal points.

- Pre-appointment, these themes relate to any thoughts, feelings or experiences prior to the medical imaging or radiotherapy appointment in question.
- During the appointment, these themes relate to any thoughts, feelings or experiences at the appointment, including experiences of the waiting room and facilities.
- Ongoing, these themes relate to any thoughts, feelings or experiences that occurred after or as a result of the appointment, and any long-lasting impacts.

Two themes did not fit within the temporal groupings, these were (i) societal beliefs, which impacts across the three time points and (ii) suggestions of solutions, which whilst not specifically asked for were issues raised by participants.

**Figure 1:**
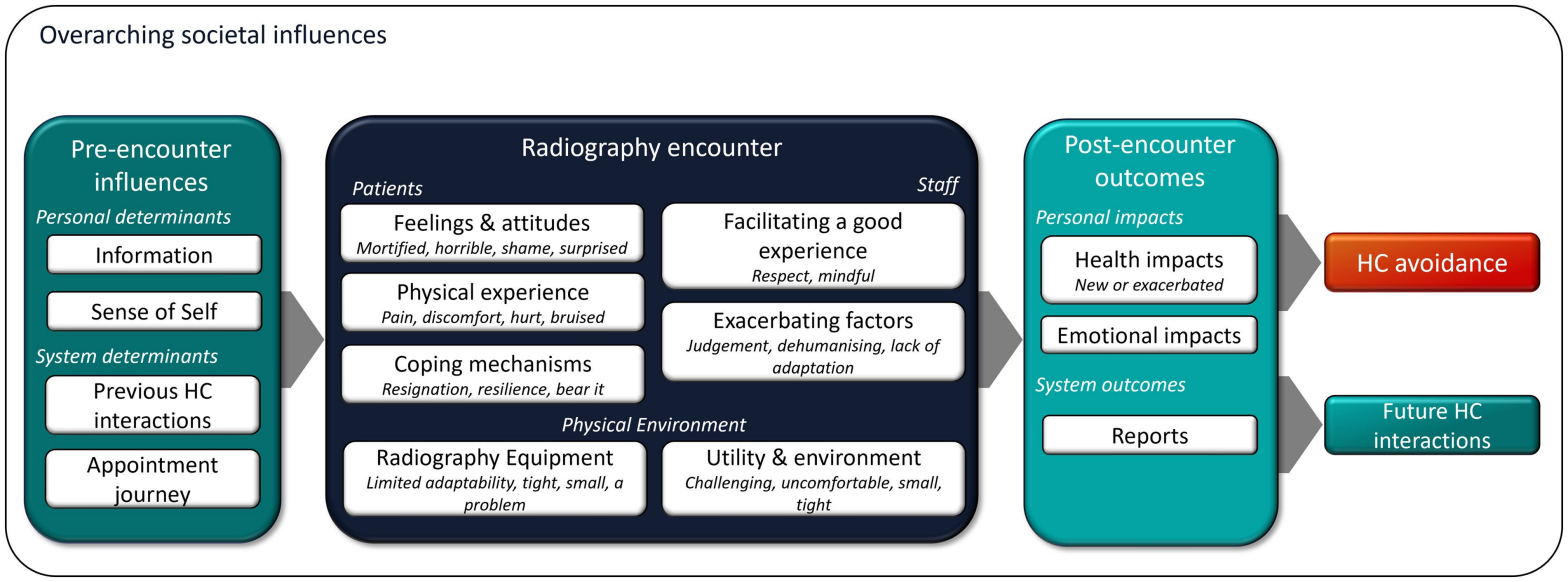
Thematic model of larger-bodied patients’ experiences of radiography. HC, healthcare.

A diagram of the themes is included in Figure 1, and indicative quotes for each sub-theme can be found in Supplementary material 2.

#### 3.1.1 Social setting and societal beliefs

As a background to all themes and time points, was both social setting and societal beliefs. There were sentiments shared that healthcare is not fit to support all people- but that neither is the world. Intersectionality was also discussed, with respondents acknowledging other privileges they held, whilst still being stigmatised for their size.

*“I think sadly whilst I am fat and therefore stigmatised in these settings, I am also a middle-class white woman who is well spoken/dressed and so I think I am often more ‘palatable’ to medicine than perhaps others are”* (P90, MI, Larger).

Some non-height defined respondents mentioned discussing issues and experiences with straight-sized friends, who could not relate, whilst others worried not only for themselves, but also for other larger or taller people.

These experiences and feelings were carried into radiography appointments with the respondents. Even those who had not experienced many health care interactions carried their experience of society in with them- in some cases preparing them for what was to come, in others potentially causing worry for experiences that did not come.

#### 3.1.2 Pre-appointment

Prior to attending appointments, it was clear that self-perceptions of the individual were influential in how they approached the health care interaction. Those with confidence in their body or appearance spoke more positively about the upcoming appointment, whilst those who identified as nervous or anxious related these feelings to the appointment content- namely scans. There were also instances where the fears about the appointment, i.e., not being able to fit in the scanner, led to unhealthy and potentially harmful actions such as extreme dieting. Such actions resulted in negative physical and mental impacts.

*“I was desperate not to have a problem so went on yet another diet. I lost about [X] pounds, before having trouble with low blood sugar and putting the weight back on. It definitely set off a period of disordered eating for me.”* (P80, MI, Larger).

Some of these considerations of the appointment were related to previous experiences either in healthcare generally or specifically in radiography. Whether the experience was positive (usually relating to their body not being an issue) or negative, the next appointment was perceived to be likely to follow suit.

*“Already feeling highly sensitive to [my] weight/body size due to [a] Dr blaming infertility on weight*.” (P49, MI, Larger).

Several of the past experiences mentioned related to what healthcare staff had said to patients about their weight or body. Healthcare services also impacted the experience of patients prior to their appointment, namely due to delays and perceived lack of planning for those with larger bodies. One respondent cited delays while the multidisciplinary team decided whether they thought she would ‘fit’ in the scanner, whilst another discussed the delay for their scan due to waiting for the larger MRI scanner to be available. The lasting impacts of such delays are discussed later.

Respondents on occasion mentioned attempting to pre-empt issues by information seeking, either through online resources or by calling ahead to check scanner capacities. Only one respondent mentioned any contact from the service to ask/check their weight in advance. However, a number of non-height defined respondents mentioned communications about risks associated with ‘higher weight’ e.g. getting hot in the MRI scanner and poorer reliability of imaging.

*“Prior to [the] appointment I was sent a standard form to complete which asked for height/weight and when I confirmed the appointment by phone I was asked if I was over 28 stone*.” (P42, MI, Larger).

#### 3.1.3 During the appointment

Commentary on the appointment itself was the largest theme, mainly as this is what the survey questions targeted. Patient experience was diverse, though overwhelmingly negative.

Participants discussed feelings and attitudes about their appointment, with the majority of them being negative. Feelings of being ‘mortified’, ‘angry’ and ‘shamed’ were frequently mentioned by those recalling medical imaging experiences. Further, discussion of being stressed, panicked or having high anxiety were also frequent, illustrating the mental toll on patients.

*“It’s very difficult not to panic that you are going to get physically stuck when one big breath would have pretty much sealed the MRI machine around me*.” (P83, MI, Larger).

Neutral and positive responses were primarily in relation to there being no issue or mention of their size or being ‘pleasantly surprised’ when fears about issues or treatment did not materialise. Neutral and positive experiences were also more commonly reported by those describing their radiotherapy treatment.

Alongside the emotional and mental experience, the physical experience of patients was discussed. These experiences spanned from comfort to pain.

Much of the physical experience was described in relation to the equipment, such as the sizes of tables, bores and coils, causing discomfort and pain in ‘fitting’ and holding ‘contorted’ positions in order to undertake the scan. In particular, for those who were taller, long scans with body parts unsupported led to ‘discomfort’, ‘numbness’ and ‘muscle tension’.

In the face of technology limitations, some staff exacerbated the pain experienced through manipulation of injured limbs, ‘rough’ handling or, in the case of ultrasound increasing the pressure applied through the probe.

*“the person insisted they also try an external scan & actually pressed so hard with the wand that they hurt me & made the delicate skin under my belly apron bleed”* (P8, MI, Larger).

Some participants discussed the coping mechanisms they undertook in response to the mental and physical impacts described. This is linked with the pre-appointment theme of self-perception. Some participants were resigned to their experiences, no longer upset as they had come to expect the treatment, whilst others’ personalities helped them feel unbothered.

*“I did not feel particularly put out as I am used to there being no allowances for my height in medical settings.”* (P78, MI, Taller).

Others felt they had little choice but to ‘grin and bear it’, motivated by the desire to get the experience over with.

The actions, communication and perceived feelings of staff were discussed at length by participants. These were either viewed as (i) exacerbating issues, (ii) facilitating a good experience, or (iii) generally neutral/minimal expected care.

Whilst we do not have the feelings and attitudes of staff directly, participants reported their perceptions of staff feelings and attitudes. Where these were perceived as negative, they exacerbated the respondents’ own feelings about themselves and their appointments. Respondents felt that their body was a nuisance or in the way for staff, that staff judged or blamed them, making the staff annoyed. Some also perceived that the staff were embarrassed to be dealing with the patient’s size, which on occasion came across as inexperience, worrying respondents.

*“I felt demeaned and that the nurses were inexperienced in dealing with sensitivities around weight and being weighed”* (P82, MI, Larger).

These perceptions were illustrated by reports of what and how staff communicated to patients, with one respondent being referred to as a ‘big one’ in front of a full waiting room. Others described feeling patronised, or stupid by the way staff communicated, whilst others shared that they were often talked about, but not to. In contrast some respondents talked about a lack of communication, with staff offering no explanations or reassurances about processes or offers of adaptations.

*“she was silent aside from huffing like it was some kind of huge effort for her…she never spoke to me all the time she was putting it on which made me feel uncomfortable and like I was some kind of burden. She went back to the computer area and said to the other woman “it will have to do” and sighed again.”* (P39, MI, Larger).

The lack of communication was linked with a feeling that there were not enough adaptations offered. Respondents who stated they told staff about pain or discomfort were frequently told there was nothing that could be done other than stopping the scan. Others felt that when there were adjustments possible, staff chose not to use them.

*“The radiographer had no interest in trying to adjust the machine to a comfortable height.”* (P54, MI, Taller).

Where staff did intervene, such as to reposition or adapt, respondents describe this being done with expressions of exertion such as ‘huffing’ making them feel like a ‘nuisance’.

Whilst not as common, there were occurrences where staffs’ attitudes and actions had a positive impact on the patient experience. Staff that were reported as respectful or kind were described, indicating empathy with patients as they apologised for limitations of equipment and services. Respondents also discussed the language and communication by staff, indicating that it was preferred when staff mirrored patient language.

*“There was some caution over the use of language by the staff, until I used appropriate words that meant I was OK with discussing it. They then engaged and reflected that language back.”* (P61, MI, Taller).

Respondents reported some instances that were interpreted as neither positive nor negative, rather that there was no difference, or basic expectations of care were met. These included mentions of staff being quick and efficient, as well as there being no mention or discernible impact of their size on their experience.

Where consequences and adaptations were noted by patients due to their size, these were often related to equipment or positioning. Some were described as matter of fact, e.g., requiring a different coil, whilst others were described as making the respondent feel an inconvenience or making the radiographers’ job harder.

*“it was obvious my size was making it more difficult for them, and I felt like I was an inconvenience and making their job harder”* (P9, MI, Larger).

For others, the consequence of their size was scans having to be repeated later by a more experienced or senior practitioner.

The imaging environment and equipment were also a large theme that impacted respondents. Whilst there was some mention of radiotherapy equipment, these comments were generally neutral or positive, with respondents describing little impact on their experience.

Generally, the lack of adjustability was discussed. This impacts both taller individuals and wider individuals across a range of modalities. The size of beds, room set up, and devices such as those used to measure height were all mentioned as being limiting in their lack of range.

*“I do not believe all Xray tables accommodate people with larger bodies”* (P18, MI, Larger).

MRI was a frequently named modality primarily relating to the bore size (the internal diameter of the scanner where the patient lays). Whilst some mentioned claustrophobia, the majority discussed being ‘squeezed in’, touching the sides or feeling as if they would get stuck and be unable to get out. The discomfort patients felt was often exacerbated by the length of protocols, what seemed a reasonable adjustment became uncomfortable or even painful as time progressed.

*“My arms hung off the side of the MRI table (I’m broad), but they needed to be by my side on the table. The only way I could do this was to kind of squeeze myself together and sit on my hands. Initially it was OK but after 10 min I needed a break and to reset. I did not get a break - they just thundered on.”* (P63, MI, Taller).

Ultrasound was another modality that was mentioned frequently, particularly for those referred for gynaecological and gastrointestinal imaging. Respondents described the additional pressure of the ultrasound probe against the skin that was applied by practitioners and how this caused discomfort and pain.

When the patients discussed imaging equipment, there was an evident knowledge gap as to how it worked. They desired ‘bigger’ and ‘stronger’ ultrasound probes (thinking that bigger probes would mean a stronger signal) or questioned why MRI bores are not just ‘bigger’. These questions left larger-bodied patients feeling as if the world was not designed for them.

*“It made me frustrated that these things aren’t just much larger in general, because if I can barely fit, what about people bigger than me?*” (P27, MI, Larger).

These sentiments were also shared in relation to utility and the environment. Respondents reported their size impacting their experience from the point of entering clinical spaces, including mentions of toilets, waiting room seating and changing rooms. The availability of appropriately sized gowns was also mentioned, often leading patients to have to be in embarrassing situations in front of other patients and clinicians.

*“The gown was way too small, so I asked if they had anything larger. They did not and the HCA* [Health Care Assistant] *had to walk closely behind me so that everyone did not see my bare back and underwear*.” (P15, MI, Larger).

#### 3.1.4 Post appointment/on-going

Radiography experience had on-going impacts on patient health. Some caused new injuries, such as bruising and wounds from the pressure of ultrasound probes. Others had existing health conditions exacerbated either by delays in imaging, or through unclear imaging.

*“Second attempt at the MRI was 5 weeks later (delaying my diagnosis of a brain tumour) in a centre with a wider machine.”* (P97, MI, Larger).

Alongside the physical, patient feelings were also impacted in the long-term. Ongoing negative feelings such as anxiety and judgement were frequently mentioned. Respondents talked about needing to seek support after their experiences including trauma informed therapy such as Eye Movement Desensitisation and Reprogramming (EMDR) therapy. These continuing negative feelings are linked with a large theme of healthcare avoidance. Many respondents indicated that they would actively avoid general medical interactions, and specifically ‘scans’ where possible. They reported ‘putting off’ investigations due to physical experiences such as being ‘squeezed’ into scanners, as well as the judgement felt. One participant even indicated that they no longer would aim to get pregnant again due to their experience, thereby avoiding the healthcare interactions that come with pregnancy.

*“My general (unconscious) approach to seeking medical advice is avoid, delay and minimise as much as possible due to fear of being judged or everything blamed on my weight.”* (P9, MI, Larger).

The written results (reports) received by patients post-imaging were also raised as having an impact, specifically when they mentioned the patient habitus in ways that had not been discussed during the appointment. A frequent mention was that body habitus had had an impact on image quality, with respondents feeling unhappy they had not been made aware of this at the point of imaging.

*“I received a letter with a summary of the scan findings. On it, it read ‘obscured view due to maternal BMI’ [Body Mass Index]. I felt very upset that this was mentioned on the letter. I was confused why, if it is a problem, it wasn’t mentioned at the appointment.”* (P21, MI, Larger).

Some respondents mentioned raising complaints. One who had a negative experience at a private clinic was offered a free repeat scan and contrasted this with their experience at an NHS site where she was told she must have misheard the clinician and nothing more was done. Another respondent was encouraged by her General Practitioner (GP) to make a complaint, but they felt they could not see the point.

Those that reported more positive or neutral feelings after their appointment also linked these to the future, indicating reassurance that they knew they fitted certain scanners and feeling more ‘relaxed next time’.

*“It made me feel positive about future scans if they happen” (P26, MI, Larger).*

#### 3.1.5 Solutions

Whilst there were no questions specifically asking for solutions to the experiences discussed, some respondents offered these. Planning ahead was mentioned, those who are Larger Bodied know they are larger, and their referring physicians know too. Some questioned why doctors are referring patients for scans that are inaccessible due to size, whilst others took it upon themselves to find out in advance the size of scanners etc.

*“I’m much more careful about checking now and I ring ahead to ask if there’s a weight limit. I try to be very neutral in my language,* e.g.*, ‘Hi, I’m fat so can you check if I will fit in x’”* (P32, MI, Larger).

In line with this, others requested more information on scanner sizes, the experience they can expect and their options.

There were some requests made for adaptations by staff. There was a recognition that being larger, they may increase the difficulty of some assessments, however, further training could overcome the difficulties. Use of language was also mentioned, with respondents requesting more neutral or positive phrasing. One such example was how blame was phrased, whether the person or technology was at fault- the patient should not be to blame.

*“The lab tech was very apologetically and very uncomfortable about saying that I was too big to fit in the machine”* (P32, MI, Larger).

Other requests included the availability of larger auxiliary equipment including gowns as well as the request that when designing new equipment, manufacturers consider the needs of all people, not just the average.

## 4 Discussion

This study provides novel qualitative evidence on the experiences of patients living in larger bodies within UK radiography services, extending across both diagnostic and radiotherapy contexts. In doing so, it addresses several gaps in existing literature which has largely focused on single modalities, non-UK settings and weight alone rather than including individuals who are above average height. Our findings highlight persistent structural limitations, particularly in relation to equipment and utilities, that shape patient experiences. However, the results also demonstrate that staff behaviours across radiography and wider healthcare roles can either alleviate or exacerbate these challenges through their attitudes and actions.

### 4.1 Size stigma and interpersonal care

In line with published literature, the participants of this study reported experiencing stigma in relation to their size. Stigma describes physical characteristics or character traits that mark an individual as having a perceived lower social value. A stigmatised trait can lead to experiences of discrimination, feelings of low self-esteem, depression and a lower quality of life (6, 7). Within this study patients reported perceptions of staff feelings and attitudes including feeling judged and / or blamed, that their body was a nuisance. Respondents also reported staff using facial expressions and huffing to indicate increased exertion and effort when interacting with larger patients. This experience is not isolated to radiography. A literature review investigating the impact of weight stigma concluded that there is significant evidence of negative attitudes generally held by healthcare providers (21). Studies have shown that implicit and explicit anti-fat bias is prevalent among Medical Doctors (MDs), similar to most people in society. Another study showed that across specialisms, including family practice, gynaecology, orthopedics etc., a strong implicit anti-fat bias was evident in evaluations of overweight individuals as being ‘bad’ and ‘lazy’ (22). Health care professionals have also shown the belief that those who are overweight are harder to treat. In a study on physical therapists, Setchell et al. (10) found almost all participants used negative words, including “dangerous,” “risky,” “hard,” “challenging,” and “difficult” when talking about working with overweight patients. In this study, non-height defined patients reported feelings of similar burden to radiographers. It is likely that the physical nature of moving patients in physical therapy, and the need to position patients for imaging, may play a role in the interactions discussed. This is in line with reports from a study on medical imaging students perceptions of larger bodied patients, where it was reported that 42.1% said they would find working with ‘ob*se’ patients frustrating, and 7.9% indicated they would rather not image them at all, which is worrying given students represent the next generation of healthcare professionals (13). Across respondent accounts and supporting literature, size stigma in radiography emerges not as isolated interpersonal behaviour, but as an interactional signal that patients’ bodies are perceived as problematic within clinical systems.

### 4.2 Patient safety, physical harm, and clinical risk

The impact of such stigma towards larger bodied patients should not be trivialised. This paper identified clear instances of poor practice and risks to patient safety. Descriptions included patients being ‘squished’ into MRI bores whilst touching the sides, which increases the risk of heating and burns through the induction of electrical currents (23, 24). Injuries were also reported from high transducer pressure in ultrasound scenarios, such injuries are scarce in published reports or ultrasound safety discussions. Whilst operators may increase pressure slightly to reduce the distance between probe and target tissue, guidelines generally indicate ‘minimal probe pressure’ to avoid impacting diagnosis (e.g., compressing tumours to mask size) or, in obstetrics, to avoid modifying the middle cerebral artery parameters used for diagnosing pregnancy associated pathologies (25). Delays in diagnosis were also detrimental especially to non-height defined patients’ health journeys. In one situation, the lack of inclusive imaging equipment resulted in the delayed diagnosis of cancer. This is in line with the literature that suggests that BMI is positively associated with greater odds of having delayed care in the past year (OR = 1.06, *p* < 0.001) (26). Considering literature also indicates those of higher weight are less likely to be referred to diagnostic testing due to an over-attribution of symptoms to weight (27, 28), it is possible that patients have already faced delays in gaining a referral adding to the potential impact of delayed diagnosis. These findings indicate that stigma in radiographic care operates not only affectively but materially, shaping clinical practices in ways that directly impact patient safety, diagnostic timeliness, and health outcomes.

### 4.3 Healthcare avoidance

The other impact of such experiences is that of healthcare avoidance. Within this study, many respondents indicated that they, where possible, want to avoid interacting with healthcare and more specifically instances that would require scans. This is in line with literature where larger patients who perceived discrimination tend to avoid seeking routine preventive care, such as cancer screenings (29, 30). Similarities have also been noted with other forms of stigma whereby patients with COPD (Coronary Obstructive Pulmonary Disorder) and neurological conditions were found to reduce health utilisation in the face of anticipated stigma (31). It is important to recognise healthcare avoidance within the wider context of health service use, such as using Andersen’s Behavioural Model of Health Service Use (BMHSU) (32). In this model avoidance is understood as ‘underutilistion given need’ where by perceived need is balanced against barriers, in this case stigma. Revision of the BMHSU (33, 34) also reflects the cyclical/dynamic experience described in our study (Figure 1) whereby patients do not experience a healthcare interaction in isolation, but rather in a dynamic fashion leading to a cycle of healthcare avoidance. Patients learn to anticipate stigma, which results a delay or avoidance of seeking medical help, leading to advanced and difficult to treat conditions and potentially further stigma/poor experiences, which in itself reinforces avoidance (21, 27, 28). This can also impact where and how patients seek health care, as evidence by a study showing larger bodied patients linked with higher presentation in Emergency Departments due to frequent changing of their GP in an effort to avoid stigma and labels (35). Viewed temporally, participants’ experiences form a cyclical pattern in which anticipated stigma, prior harm, and system barriers accumulate over time, reinforcing healthcare avoidance rather than representing discrete negative encounters.

### 4.4 Positive practice and staff potential

Whilst this may currently seem quite a scathing review of radiographers, this work also highlighted the ability of healthcare professionals to break the cycle of poor experience and poor care, reducing patient anxiety and increasing confidence in future healthcare. This aligns with studies which indicate that providing a welcoming and less threatening healthcare environment for patients improves their experience- reducing the barriers to care (13). Themes including reducing focus on body weight, adopting patient-centric communication strategies and providing an inclusive clinic environment are indicated both in this study and the literature as having a positive impact (13). Importantly, this same cycle was shown to be interruptible, with respectful communication, visible effort to adapt care, and transparent explanations reframing encounters in ways that altered patients’ expectations of future healthcare.

### 4.5 Training, interventions, and professional development

Interventions and training are required to ensure that radiographers among other healthcare professionals are equipped with the behaviours, language and experience to provide empathetic and person-centred care to those with larger bodies (36, 37). Rather than being limited to skills-based training alone, these approaches need to address the underlying assumptions that chape clinical practice, including aspects of the medical model of ob*sity, it also extends more broadly to the construction of certain bodies as ‘other’ within clinical settings, including anyone viewed outside of the perceived ‘universal’ patient, which may which be associated with perceptions of increased difficulty, inconvenience or deviation from the ‘expected patient (35, 38). Addressing this cycle therefore requires interventions that engage both the implicit beliefs and structural assumptions held by healthcare professionals alongside their communicative and clinical practises. This includes embedding patient centred communication strategies reducing unnecessary focus on body characteristics where not clinically relevant and fostering inclusive clinical environments that minimise the positioning of certain bodies as inherently problematic or burdensome (13, 37). The enhancement of technical confidence and skills for diverse body should be core to these interventions, ensuring that healthcare professionals are not only prepared for the presumed ‘universal’ patient, but are also able to deliver appropriate adaptable an equitable care to a wide range of body types and presentations in clinical practice (13).

Furthermore, clear clinical pathways for the management of patients who are not able to be imaged using conventional equipment are required. Respondents in this study reported long delays and uncertain next steps, leading to increased anxiety. Radiography departments should explore agreements and processes for onward referral to centres with different equipment, or a clear pathway that utilises different imaging techniques that are less size constrained to ensure a timely diagnosis for potentially urgent cases.

### 4.6 Space, equipment and structural exclusion

To understand why these experiences persist beyond individual interactions, it is necessary to consider how radiographic environments, technologies, and routines are organised around assumptions of a ‘universal’ patient body. This study highlights that medical stigma extends beyond the interpersonal interactions between staff and patients and includes spaces and equipment. This may also be understood through a spatial lens, drawing on the work of Henri Lefebvre (39). Healthcare environments can be seen as comprising space (the planned and designed environment), perceived space (how space is organized and operationalized) and lived space (how it is experienced by individuals). Within this framework, participants’ accounts highlight how radiographic services are often designed round the assumption of a ‘universal’ patient body, evident in the dimensions of equipment and facilities.

Whilst it may be common knowledge to radiographers that equipment limitations are due to technological limitations, e.g., larger MRI bores make it harder to create the consistent magnetic field required for high quality images, this is not apparent to patients. These constraints reflect the level of conceived space, where design decisions (often driven by technological and engineering requirements) shape what is considered possible within imaging environments. Being able to provide an explanation for such limitations can help patients understand why some examinations need adapting or are not fully achievable, rather than, for example, finding out afterwards via an imaging report. In this sense, communication becomes part of perceived space, influencing how these constraints are encountered and interpreted in practice.

At the level of lived space, participants reflect how these structural and organizational features are felt in practice, including discomfort, uncertainty and embodied exclusion (40). There is also an argument that radiographers should regard the ability to adapt technique for patients with different sized bodies (such as being able to use non-standard coils in MRI, adapt exposure factors in general radiography), as a core skill, in the same way as they are able to modify technique for other patients, such as those who are non-ambulatory. Such adaptations can be understood as attempts to mediate between conceived and lived space, mitigating some of the limitations imposed by design.

Finally, there may be scope for equipment manufacturers to consider ancillary equipment to support a wider range of patients, e.g., extension boards to support the limbs of height-defined respondents. This points to the potential for reconfiguring conceived space itself, challenging assumptions of a standardised patient body. These findings suggest forms of embodied exclusion, whereby individuals whose bodies fall outside of normative expectations encounter structural barriers within healthcare environments (40, 41).

Taken together, these accounts illustrate forms of embodied exclusion in which interpersonal stigma, technological limitation, and spatial design converge to position certain bodies as out of place within radiographic care.

### 4.7 Strengths and limitations

One of the strengths of this paper is the inclusion of marginalized and frequently overlooked populations within this research. This was only made possible through the inclusion of PPIE representatives from the inception of the project, providing insight, advocation and direction to the study team. The involvement of PPIE representative from the point of funding supports contributors in engagement (42). As highlighted in the ‘Valuing all voices framework’ (43), concepts of trust, self-awareness, empathy, and relationship building are particularly key when engaging with social justice and health equity lens- as with this work focused on. Further information and discussion on the PPIE approach utilised in this study can be read in Hancock et al. (18).

A further under-represented population were those who identified as height-defined larger bodied patients. At present there are no peer reviewed articles to the authors’ knowledge who specifically examine the experiences of taller people in radiography, in fact there is very little literature on experiences of healthcare in general. Most papers that do touch on this topic look at quantitative aspects such as care outcomes (44–46), with a paucity touching on lived experiences. Height-defined participants in this study faced similar physical barriers to their larger counterparts, citing similar issues with gowns, chairs, changing rooms, imaging and treatment equipment. However, their experience of staff generally included fewer negative connotations. Respondents mentioned occurrences of hilarity, or their height being seen as a joke. They generally took this well, however, reported it impacting their feeling of belonging- indicating they felt the world was not made for them. This aligns in part with the limited literature on the experiences of height-defined individuals in everyday contexts. Whilst there is some overlap between the experiences of height-defined and non-height-defined larger bodied individuals (namely barriers to equipment and auxiliary), the distinct experiences of height-defined individuals reported here warrant further study dedicated to understanding these further.

One of the primary limitations of this study was the sample recruited. Whilst we achieved our target recruitment, only 4.4% of respondents identified as an ethnicity other than white. This underrepresentation may in part reflect the sampling approach, including reliance on researchers’ and PPIE networks for dissemination via social media, which were themselves relatively homogeneous in composition and may therefore have limited the reach of the study into more ethnically diverse populations. As, there is a well-established link between racism and anti-fat bias, those who are both non-white and living in a larger body may have different experiences, potentially worse than those who are white (47, 48). This was part of the motivation for developing a wider PPIE group with greater representation, and we recommend future research employs methods that enhance the recruitment of a range of ethnicities. Further, we did not specifically gather data on whether respondents had a disability. From our data, and PPIE input, it became apparent that those with an intersection of need faced barriers greatly limiting their care. Given the necessity for access including wheelchairs and hoists in imaging and treatment scenarios, there is further work required to understand these barriers and potential solutions.

Whilst respondents did report some neutral and positive experiences, we recognise that respondent bias and self-selection bias could have driven the widespread reporting of negative experience; however, the inclusion of accounts that also described positive encounters and expressed understanding of service pressures suggests a more nuanced dataset than a purely negative framing might imply. In addition, recruitment via online platforms and social media may have further shape the sample composition, as such channels can facilitate participation among individuals with stronger or more salient experiences of healthcare including those engaged with body size advocacy communities (49). This may have influenced the distribution of reported experiences, although the direction of this effect is not straightforward, as engagement with advocacy networks may also reflect a range of motivations and perspectives rather than uniformly negative experiences (50).

### 4.8 Conclusion

This study highlights substantial shortcomings in radiographic care for people with larger and taller bodies. Participants described stigmatising attitudes, inaccessible environments and unsuitable equipment, with clear implications for safety and timely care. Positive encounters, however, showed that staff can disrupt these patterns, reduce anxiety and improve confidence. Services would benefit from auditing equipment and facilities to identify gaps in provision, developing clear pathways for patients who cannot be imaged with standard approaches, and strengthening training for staff and students to challenge assumptions about larger patients. Clear information for patients on available services and potential limitations is also needed.

Further research should explore the experiences of taller individuals, whose needs remain largely overlooked. The limited ethnic diversity of the sample indicates the importance of recruiting participants from a broader range of backgrounds, particularly given the intersections of racism and disability. Overall, the findings emphasise the need for equitable, safe and respectful radiography care for all body types.

## Data Availability

The raw data supporting the conclusions of this article will be made available by the authors, without undue reservation.
