# Supplementary material for: Navigating radiography as a larger-bodied patient: a qualitative exploration
Source: Front Public Health. 2026 Jun 15;14:1803012. doi: 10.3389/fpubh.2026.1803012 (PMC13310734; doi:10.3389/fpubh.2026.1803012)
Supplement: Supplementary file 1 [file Data_Sheet_1.DOCX]

Supplementary Material 1

Questionnaire: questions relating to experiences of undergoing Medical Imaging or Radiotherapy.

**MEDICAL IMAGING EXPERIENCE**

24. Can you tell us whether having a larger body impacted on your experiences of the imaging procedure, what occurred, and how you felt about it? (e.g. how it was carried out, any adaptations made and non-completed scans? [FREE TEXT RESPONSE]

25. Can you tell us whether having a larger body impacted on your experiences of staff attitudes during the imaging procedure and how this made you feel? If this does not apply to you, please type NA. [FREE TEXT RESPONSE]

26. Can you tell us if / how your experience of the scan and staff attitudes impacted your longer-term attitudes to and interactions with healthcare? [FREE TEXT RESPONSE]

27. Please indicate whether you feel the availability of general equipment and accessories (such as waiting room chairs, gowns etc.) was impacted in some way due to your body size and / or shape?  [FREE TEXT RESPONSE]

28. For each of the following pieces of equipment, please indicate whether you feel the availability of suitable equipment was impacted in some way due to your body size and / or shape? [TICK BOX RESPONSE]

- Waiting room chairs
- Hospital beds
- Hospital gowns
- Weight measurement devices e.g. scales
- Height measurement devices
- Changing rooms
- Toilet facilities
- Other (FREE TEXT)

29. Thinking of the instances above, can you please tell us how your shape and/or size impacted on your experience of needing, and receiving appropriate equipment? [FREE TEXT RESPONSE]

30. When you attended for your Medical Imaging appointment / procedure, please indicate whether you feel the design of the imaging equipment, including accessories, impacted upon your experience due to your body size and / or shape?

- Yes [IF YES – TO QUESTION 31]
- No [IF NO – TO QUESTION 32]

31. Please describe the situation and how it impacted on your feelings, emotions and experiences of the healthcare situation? [FREE TEXT RESPONSE]

32. Is there anything else you want to tell us about your experience of medical imaging while having a larger body? [FREE TEXT RESPONSE]

RADIOTHERAPY EXPERIENCE

33. Thinking of your radiotherapy experience, which part/s of your body/organ was the radiotherapy given to? [FREE TEXT RESPONSE]

34. Can you tell us whether you believe having a larger body impacted on your experience of radiotherapy **planning**. If so what occurred, and how did you feel about it? [FREE TEXT RESPONSE]

35. Can you tell us whether you believe having a larger body impacted on your experience of radiotherapy **treatment**. If so what occurred, and how did you feel about it? [FREE TEXT RESPONSE]

36. Can you tell us whether you believe having a larger body impacted on the radiotherapy staff attitude(s) towards you during your radiotherapy **planning**. If so what occurred and how did you feel about it? [FREE TEXT RESPONSE]

37. Can you tell us whether you believe having a larger body impacted on the radiotherapy staff attitude(s) towards you during your radiotherapy **treatment**. If so what occurred and how did you feel about it? [FREE TEXT RESPONSE]

38. Where any moveable accessories used during your radiotherapy treatment (such as vacuum bags, thermoplastic shells, cushions)?

- Yes [IF YES – TO QUESTION 39]
- No [IF NO – TO QUESTION 40]

39. Which of these accessories were used during your radiotherapy treatment? Please tick all that apply. [TICK BOX RESPONSE]

- Vacuum bag (a big, moulded cushion from which the air is removed to shape around your body).
- Thermoplastic head shell (a mould of your face/head placed over your head and/or neck during treatment).
- Thermoplastic chest shell (a mould of your chest placed over your chest during treatment).
- Thermoplastic pelvis shell (a mould of your pelvis placed over your chest during treatment).
- Thermoplastic limb shell (a mould of your arm or leg placed over your arm or leg during treatment.
- Breastboard (laid upon during your treatment, usually with your arms up and holding on to poles).
- Lung board ((laid upon during your treatment, usually with your arms up and holding on to poles).
- Knee cushion (placed under your knees during treatment).
- No equipment but you had to lay prone (face down on your front).
- Not sure.
- Other (FREE TEXT)

40. Could you please tell us about your experience of these accessories being used and whether you felt they were appropriate for your shape / size? [FREE TEXT RESPONSE]

41. Did you need to lay prone (face down on your front) for your radiotherapy procedure?

- Yes [IF YES – TO QUESTION 42]
- No [IF NO – TO QUESTION 43]

42. Could you please tell us about your experience of laying down on your front during your radiotherapy procedure? [FREE TEXT RESPONSE]

43. When you attended for your radiotherapy, please indicate whether you feel the design of the radiotherapy equipment (the radiotherapy machines) impacted upon your experience due to your body size and / or shape?

- Yes [IF YES – TO QUESTION 44]
- No [IF NO – TO QUESTION 45]

44. Please describe this situation and how it impacted on your feelings, emotions and experiences of the healthcare situation? [FREE TEXT RESPONSE]

45. Is there anything else you want to tell us about your experience of radiotherapy while having a larger body? [FREE TEXT RESPONSE]
