# Supplementary material for: Navigating radiography as a larger-bodied patient: a qualitative exploration
Source: Front Public Health. 2026 Jun 15;14:1803012. doi: 10.3389/fpubh.2026.1803012 (PMC13310734; doi:10.3389/fpubh.2026.1803012)
Supplement: Supplementary file 2 [file Table_1.DOCX]

Supplementary Material 2

# Supplementary Data

| **Theme** | **Example (Participant number, medical imaging (MI), larger or taller)** |
| --- | --- |
| ***Social setting and societal beliefs*** | |
| Not fit to support all people | *‘This caused a problem for 6'2" me. Goodness knows what my 6'5" friend would have done!’ (P62, MI, Taller)*  *‘The system is not fit for the purpose of supporting all people.’ (P85, MI, Larger)* |
| Discussions with others | *‘I always describe to friends how hard radiologists press down on pelvic US but my slim friends don't understand as they don't suffer that’ (P6, MI, Larger)* |
| Societal norms | *‘It is an eternal issue around being tall in a world not designed to be inclusive.’ (P61, MI, Taller)*  *‘I think sadly whilst I am fat and therefore stigmatised in these settings I am also a middle-class white women who is well spoken/dressed and so I think I am often more ‘palatable’ to medicine than perhaps others are’ (P90, MI, Larger)* |
| ***Pre- appointment*** | |
| Self-perceptions |  |
| Confident | *‘I don't have an issue with my body type, I am tall so can pull off being fat (big belly as well) at 2m tall I am a confident person’. (P53, MI, Larger)* |
| Nervousness or Wary | *‘I think I get anxious before that I'm too heavy or won't fit in machines’ (P26, MI, Larger)*  *‘I felt anxious about being too big for the scanner.’ P81, MI, Larger)* |
| Experiences of or desires for the smaller self | *‘This was not an issue when I had scans many years ago. At that time my body shape was much smaller.’ (P85, MI, Larger)*  *‘I was desperate not to have a problem so went on yet another diet. I lost about six pounds, before having trouble with low blood sugar and putting the weight back on. It definitely set off a period of disordered eating for me.’ (P80, MI, Larger)* |
| Healthcare Service |  |
| Delays | *‘I had to wait 4 weeks while my mdt decided that they didn’t think I would fit in the MRI scanner but would send me anyway “as a trial” (P24, MI, Larger)* |
| Lack of planning | *‘They had to reschedule me to a large machine despite knowing my size beforehand.’ (P13, MI, Larger)* |
| Previous experience |  |
| Negative attitudes of staff in previous healthcare experiences | *‘I felt quite numb to it because I have experienced fatphobia in every medical setting for years.’ (P27, MI, Larger)*  *‘I was apprehensive about the ultrasound after negative experiences around my weight in healthcare’ (P55, MI, Larger)* |
| Positive previous experiences | *‘I had been fortunate to not have issues regarding my size in the past’ (P94, MI, Taller)* |
| Information Seeking |  |
| Patient information seeking | *‘I was apprehensive about the ultrasound after negative experiences around my weight in healthcare, and reading online ultrasounds can be harder to perform for the technician with belly fat.’ (P55, MI, Larger)*  *‘I did phone first to make sure it had the necessary weight capacity.’ (P8, MI, Larger)* |
| Provided information to patients | *‘All the pregnancy paperwork talks about poor reliability if high BMI, although it was fine’ (P17, MI, Larger)* |
| HC seeking information from patients | *‘Prior to appoinnment, I was sent a standard form to complete which asked for height/eight and when I confirmed the appointment by phone I was asked if I was over 28 stone.’ (P42, MI, Larger)* |
| ***During appointment*** | |
| Patient experience |  |
| Coping Mechanisms |  |
| Resignation | *‘I did not feel particularly put out as I am used to there being no allowances for my height in medical settings.’ (P78, MI, Taller)* |
| Resilience | *I’m a bit imperturbable so I don’t really care. It doesn’t bother me.*  *(P1, MI, Larger)* |
| Get it over with | *grin and bear it (P78, MI, Taller)*  *‘I tried to just zone out but felt upset all the way through and just wanted it over with.’ (P39, MI, Larger)* |
| Feelings or attitudes |  |
| Negative | *‘the day arrived I was so mortified that I was only there to see if I could “fit” into the machine… everyone else knew I was only there for that reason too’ (P24, MI, Larger)*  *‘It made me feel horrible about myself, like they were looking at a whale, not a human.’ (P36, MI, Larger)*  *‘Makes me feel angry that medical staff can treat us with so little respect.’ (P48, MI, Larger)*  *‘It’s very difficult not to panic that you are going to get physically stuck when one big breath would have pretty much sealed the MRI machine around me.’ (P83, MI, Larger)*  *‘I was made to feel a nuisance & left feeling shamed and sore.’ (P8, MI, Larger)*  *‘Increased anxiety about whether I would fit in the machine to have important scans.’ (P13, MI, Larger)* |
| Neutral | *‘I am fat, BMI 35, but no issue with my X-ray and other fat patients in the waiting too’. (P44, MI, Larger)* |
| Positive | *‘[I read] online ultrasounds can be harder to perform for the technician with belly fat. I was pleasantly surprised that this wasn’t presented as an issue to me’ (P55, MI, Larger)*  *‘…in a centre with a wider machine. The experience was completely different- all staff were friendly and welcoming and if there were any comments made about my size or the suitability of the machine, I didn’t hear any of it.’ (P97, MI, Larger)* |
| Physical |  |
| ‘Comfort’ | *‘(in the wider machine) I was comfortable throughout the procedure’ (P97, MI, Larger)*  *‘Machines were only adjustable to a certain point, as a tall and curvy person, I felt resigned to being as ‘comfortable’ as possible to get the procedure done.’ (P2, MI, Larger)* |
| Discomfort |  |
| Mild discomfort | *‘I was one tucked into an MRI... Squeezed in so I could feel it around me.’ (P13, MI, Larger)*  *‘There were attempts to check I was comfortable but as above, no advice or support other than to stop when I expressed discomfort.’ (P2, MI, Larger)* |
| High discomfort | *‘It was always uncomfortable as they wanted me to hold my apron belly up and some people pushed into me really hard.’ (P11, MI, Larger)*  *‘My legs were too long and I spent the 1.5hr long scan cold from my legs sticking out, and uncomfortable as my hands had gone numb from being up against the side of the machine.’ (P2, MI, Taller)*  *‘I was squeezed in quite tight which became rather uncomfortable to manage especially with my chronic pain.’ (P27, MI, Larger)*  *‘it felt like quite a tight fit, and I had to get my arms into an uncomfortable position as they wouldn't fit at my sides.’ (P4, MI, Larger)*  *‘When I have a transthoracic echo, the couch, both its whole length and especially the top elevated part have been nowhere near long enough, leading to discomfort, muscular tension, difficulty supporting my head.’ (P76, MI, Taller)* |
| Pain |  |
| From equipment | *‘And trying to put a small to me brace/thing over my knee. But it didn't fit, so had to be bodged and it was very tight and painful’ (P31, MI, Larger)*  *‘I experience not just the discomfort from the procedure but knee and back pain from contorting my body to fit the machine’ (P51, MI, Taller)* |
| From staff | *‘I didn't fit on the table properly, forcing the staff to manipulate my leg, which was severely broken. This caused a lot of pain but, thankfully, I was ripped to the tits on weapons-grade morphine.’ (P77, MI, Larger)*  *‘The last time, I booked just a trans-vaginal scan but* *the person insisted they also try an external scan & actually pressed so hard with the wand that they hurt me & made the delicate skin under my belly apron bleed.’ (P8, MI, Larger)*  *‘They dug the probe into my stomach really hard and very far to get a good enough picture and it was painful and I ended up sore and bruised for several days after.’ (P9, MI, Larger)* |
| Positioning and placement | *‘The person adjusting the x ray machine that revolve around my head was too short to reach my hight. I had to stand still in a half crouch position for X-ray which was extremely uncomfortable and hard to keep still’ (P54, MI, Taller)*  *‘Stomach over hang made placing the marker tattoos harder.’ (P57, Radiotherapy, Larger)*  *‘Feet hanging off the end and interfering with other equipment in the room’ (P78, MI, Taller)* |
| Rough handling | *‘they need to check how full my bladder was with ultrasounds, and some nurses would be a bit rougher because of “my body fat” in that area.’ (P16, Radiotherapy, Taller & Larger)*  *Handling by operator was rough to say the least. (P62, MI, Taller)* |
| Tight squeeze | *‘I was one tucked into an MRI... Squeezed in so I could feel it around me.’ (P13, MI, Larger)*  *‘my hands had gone numb from being up against the side of the machine.’ (P2, MI, Larger*  *‘having to use a larger machine and even then my body was touching the sides’ (P93, MI, Larger)* |
| Staff |  |
| Exacerbating the issue |  |
| Negative perceived feelings of staff (annoyance, blame, body in the way, judgement, inexperience, minimising, rude) | *‘The member of staff carrying out the ultrasound was cold and appeared annoyed/frustrated that she couldn't always get a good image first time.’ (P7, MI, Larger)*  *‘The administrators attitude, body language and tone of voice made it very clear that I was an issue for her, and that was before I even walked in to the clinical area.’ (P97, MI, Larger)*  *‘felt to blame when measurements couldn’t be obtained.’ (P95, MI, Taller)*  *‘The technician was embarrassed that I didn't fit. I wish he could have just been neutral about it.’ (P32, MI, Larger)*  *‘I felt some sense of anger about feeling like I was being viewed as a problem again’ (P27, MI, Larger)* |
| Negative communication (dehumanise, commented on, lack of communication, talked about, patronised) | *‘she threw the wand down and declared "there's no point carrying on with you like this."’ (P11, MI, Larger)*  *‘Staff always speak to you like you are an idiot when you are fat’ (P15, MI, Larger)*  *‘The clinician came to take me through but as he did he announced to the group of colleagues in front of him and a waiting room full of people behind him that they “had a big one” not my name instead he chose to dehumanise me at a major oncology hospital that I have to trust for the rest of the time I have left.’ (P24, MI, Larger)*  *‘there were discussions had about me within earshot without speaking to me’ (P92, MI, Larger)* |
| Lack of adaptations | *‘No adaptations were made or offered and I know they can offer scans via the vaginal canal but this wasn’t offered to me.’ (p12, MI, Larger)*  *‘I did tell the radiographers however, they said they couldn’t do anything and to wait it out or stop if needed’ (P2, MI, Larger)*  *‘The radiographer had no interest in trying to adjust the machine to a comfortable height.’ (P54, MI, Taller)* |
| Unable to complete scan or aborted scan | *‘The scan had to be aborted because I didn't fit in the MRI scanner. I had already been cannulated and given medication which was annoying.’ (P32, MI, Larger)*  *‘They had to reschedule me to a large machine despite knowing my size beforehand.’ (P13, MI, Larger)* |
| Exertion | *‘They huff etc, it makes you feel a nuisance.’ (P31, MI, Larger)*  *‘During this she was silent aside from huffing like it was some kind of huge effort for her’ (P39, MI, Larger)* |
| Alleviating or facilitating a good experience |  |
| Positive perceptions of staff attitudes (respect, apologetic, mindful) | *‘The staff on the day were very respectful and kind to me’ (P67, MI, Taller)*  *‘the female sonographer made me feel secure, she was direct but her choice of language was polite - acknowledging that everyone has different body shapes and that isn't an issue or hindrance.’ (P94, MI, Taller & Larger)* |
| Positive communication (humour, reflection pt language) | *‘Some good-natured banter but otherwise they were very professional throughout.’ (P77, MI, Taller & Larger)*  *‘There was some caution over the use of language by the staff, until I used appropriate words that meant I was OK with discussing it. They then engaged and reflected that language back.’ (P61, MI, Taller & Larger)* |
| General neutral/made no difference |  |
| Efficient and focussed on the job | *‘Imaging staff were quick and efficient getting the scans done and I personally didn't have any issues’ (P34, MI, Larger)*  *‘I think they went into problem solving mode rather than had an impact on their attitude.’ (P14, MI, Larger)* |
| Neutral perceptions of staff attitudes | *‘Staff were lovely and didn’t make an issue.’ (P57, Radiotherapy, Larger)*  *‘Staff didn't make me feel uncomfortable, weight was never mentioned at the appointment.’ (P42, MI, Larger)* |
| Consequence and adaptations |  |
| Adaptation attempts | *‘it was obvious my size was making it more difficult for them and I felt like I was an inconvenience and making their job harder when it should be when they were obviously struggling to get the view they wanted and trying different places, getting me to move and then trying the original place again and digging the probe in further’ (P9, MI, Larger)*  *‘Once I'd laid down she said she needed to put a cage around my knee. She got it and tried to close it on my knee but it wouldn't fit… She got another device but not before trying to force the smaller one on me again.’ (P39, MI, Larger)* |
| Senior/experiences staff intervention or repeats | *‘In both pregnancies my 20w scans had to be completed a week later by a senior member of staff.’ (P11, MI, Larger)* |
| Environment and equipment |  |
| General | *‘Machines were only adjustable to a certain point’ (P2, MI, Larger)*  *‘Not everyone can lie flat especially larger frames and need pillows or support under body’ (P29, MI, Larger)*  *‘Being tall and the machine not adjusting if you are above average.’ (P62, MI, Taller)* |
| MRI | *‘My height caused a problem with an MRI scanner. Whole body imaging requested but the platform could not withdraw sufficiently without hitting cupboard at rear.’ (P41, MI, Taller)*  *‘It was incredibly tight in the MRI scanner, my arms were getting stuck along the sides of the machine as there just wasn’t enough space.’ (P83, MI, Larger)*  *‘I had to have a brain MRI and the staff were great and tried to get me into the scanner but the bore was too narrow.’ (P14, MI, Larger)* |
| Ultrasound | *‘the technician pushing VERY hard on my stomach. I’ve had several ultrasounds prior to this in a previous pregnancy when I was several dress sizes smaller and never experienced such force used.’ (P12, MI, Larger)*  *‘they always wrote that it wasn't clear because of my body, whereas they should be able to accommodate different depths of abdominal fat.’ (P80, MI, Larger)* |
| Radiotherapy equipment |  |
| Utility and environment |  |
| Transfers | *‘I am an amputee so manoeuvring onto a scanner from a wheelchair when I am (was) 100kg is challenging.’ (P41, MI, Larger)*  *‘I struggled to climb the steps to get onto the machine.’ (P81, MI, Larger)* |
| Gowns | *‘Gowns are never available in the right size. Neither are paper underwear do it's uncomfortable from the off.’ (P13, MI, Larger)*  *‘The gown was way too small so I asked if they had anything larger. They did not and the HCA had to walk closely behind me so that everyone did not see my bare back and underwear.’ (P15, MI, Larger)* |
| Chairs | *‘Seating - all waiting rooms need some higher seated chairs.’ (P61. MI, Taller)  ‘For me it is the stuff around the imagining- the changing rooms, the gowns, the waiting chairs’ (P15, MI, Larger)* |
| Changing rooms and loos | *‘The changing rooms were tight and so I was uncomfortable getting changed’ (P3, MI, Larger)*  *‘I was told to undress and put on a gown in a very small changing area (this was a portable MRI in a portacabin), I felt this was hard due to my size and pain but appreciated only so much space is available in these kinds of scanners.’ (P39, MI, Larger)*  *‘the changing room was woefully small for my longer limbs and changing was difficult’ (P60, MI, Taller)* |
| **Ongoing** |  |
| Patient Health |  |
| Caused new health issues | *‘I ended up sore and bruised for several days after.’ (P9, MI, Larger)* |
| Impact on diagnostics and treatment | *‘I had to wait 4 weeks while my MDT decided that they didn’t think I would fit in the MRI scanner but would send me anyway “as a trial” …Needless to say me and my tumour did not fit in the MRI so I was sent home with still no answers and further delaying my cancer diagnosis. 8 weeks after my “trial” and 8 hour debulking surgery I was told my ovarian and womb cancer was too advanced to cure.’ (P24, MI, Larger)*  *There were question marks over my PCOS diagnosis for a long time (despite meeting all other diagnostic criteria) because they couldn’t get a clear view of my ovaries due to my “DBH” - which I later found out meant difficult bodily habitus (or similar) - a medical synonym for fat.’ (P28, MI, Larger)*  *‘Second attempt at the MRI was 5 weeks later (delaying my diagnosis of a brain tumour) in a centre with a wider machine.’ (P97, MI, Larger)* |
| Patient Feelings |  |
| Negative (confusion, worry, fear, anxiety, resignation, stigma, trauma) | *‘I still have anxiety every time I step foot in the hospital (and I’m there a lot) wondering if I’m going to be treated as a human being or an animal.’ (P24, MI, Larger)*  *‘I’ve been made to feel like it’s not enough and if my investigations and treatment aren’t successful it’s because of my weight. It makes me feel less worthy of interventions.’ (P45, MI, Larger)*  *‘Long-term attitude, grin and bear it. Expect nothing to fit.’ (P78, MI, Taller)*  *‘I struggle to trust HCP’s and had to have trauma therapy (EMDR) to reprocess the trauma I have endured being a fat person going through cancer treatment and care.’ (P24, MI, Larger)* |
| Positive | *‘It made me feel positive about future scans if they happen’ (P26, MI, Larger)*  *‘My attitude to healthcare staff is unchanged, admire and respect their dedication’ (P37, MI, Larger)*  *‘I’ll be more relaxed next time’ (P19, MI, Larger)* |
| Neutral | *‘Now I know which scanner I am ok to fit into for future reference’ (P14, MI, Larger)* |
| Impact on future lifestyle choices | *‘I won't get pregnant again now’ (P11, MI, Larger)* |
| Healthcare avoidance | *‘I would actively seen to avoid scans because it is incredibly uncomfortable to be squeezed in’ (P13, MI, Larger)*  *‘My general (unconscious) approach to seeking medical advice is avoid, delay and minimise as much as possible due to fear of being judged or everything blamed on my weight.’ (P9, MI, Larger)*  *‘Put me off chasing up this particular issue, even though it still bothers me as I don't want to have those same experiences.’ (P7, MI, Larger)* |
| Reports reinforce negative attitudes | *‘I received a letter with a summary of the scan findings. On it it read ‘obscured view due to maternal BMI’. I felt very upset that this was mentioned on the letter as I was worried my weight had stopped them seeing what they needed to on the scan. I was confused why, if it is a problem, it wasn’t mentioned at the appointment.’ (P21, MI, Larger)* |
| Complaints and outcomes |  |
| NHS | *‘In my scan report she even mentioned my weight for no reason (my GP confirmed this)… My GP said I should make a complaint about her but past experiences made me wonder what the point was?’ (P39, MI, Larger)* |
| Private | *‘I also had a negative experience at a private scanning clinic with someone who basically said "I'll do my best but when you're like this..." I complained. She was agency and they were furious. I was given a free re-scan and endless apologies (please note the difference there to NHS, who when I complained basically said I must have misheard her. All bullsh*t.)’ (P11, MI, Larger)* |
| 1. **Solutions** |  |
| Planning ahead |  |
| By patients | *‘I'm much more careful about checking now and I ring ahead to ask if there's a weight limit. I try to be very neutral in my language e.g. “Hi, I'm fat so can you check if I will fit in x”’ (P32, MI, Larger)* |
| By staff | *‘I mean the Dr knew my size and I find the lack of communication between the department disappointing because she could have saved me the hassle when booking the appointment. She sends people for MRIs all the time she should know there is a possibility people in larger bodies wouldn't fit.’ (P32, MI, Larger)* |
| Information resources | *‘It would be great to have an honest and reliable resource, perhaps from the NHS, on what to expect with medical imaging as a person in a larger body’ (P55, MI, Larger)*  *‘I would appreciate if there was more information around options.’ (P30, MI, Larger)* |
| Requests for staff |  |
| Adaptations | *‘Although I appreciate that my larger body can make certain scans more difficult to assess, I still think that there can be further training on how to overcome these difficulties’ (P28, MI, Larger)*  *‘It would have been amazing if they could have paused for a break and allowed me to change position or something. Even a pillow under my legs, anything! (I had a brain scan)’ (P63, MI, Larger)* |
| Language | *‘Staff should consider their use of language and expressions.’ (P46, MI, Larger)*  *‘The lab tech was very apologetically and very uncomfortable about saying that I was too big to fit in the machine/the machine was too small to accommodate me. I wish he could have used more neutral body language.’ (P32, MI, Larger)* |
| Larger auxiliary equipment (e.g. gowns) | *‘I would appreciate if there was ... Larger gowns, machine sizes etc.’ (P30, MI, Larger)* |
| Design of equipment | *‘In designing future equipment manufacturers need to be cognoscente of body size - A longer bed and a wider tube are INCLUSIVE of all people, whereas the current equipment sometimes feels to me like it is being worn by me, rather than used....’ (P61, MI, Larger)* |
