# Supplementary material for: Navigating radiography as a larger-bodied patient: a qualitative exploration
Source: Front Public Health. 2026 Jun 15;14:1803012. doi: 10.3389/fpubh.2026.1803012 (PMC13310734; doi:10.3389/fpubh.2026.1803012)
Supplement: Supplementary file 3 [file Table_2.DOCX]

Supplementary Material 3

# Researcher Debrief Guide

**Aim of guide:**

To provide a structure / resource for research project members contacted via study participants.

1. Contact will be via email. We do not suggest researchers offer phone calls as researchers do not have training in offering mental health support or first aid.
2. To determine the best resources to signpost the study participant towards, this guide uses the ‘what, so what, now what’ framework. Researchers should use their best judgement on how to signpost participants without probing for further information. The table below gives some examples but is not definitive and has not been prepared by a mental health or wellbeing practitioner. The researchers may update / add resources to this guide as the study progresses.
3. To support researchers, a draft email text is provided at the end of this document.
4. As a way of monitoring the use of this debrief guide a log of queries / concerns and the signposting resources used will be kept (see penultimate page of this document). However, NO identifying information will be recorded other than the name of the researcher and the date the resources were provided.

| **What?**  What emotions / feelings is the study participant describing? What is the reason they’ve reached out for support? | **So What?**  What impact is the study participant’s current emotional state having on them?  If this is not specified, send all related resources. | **Now What?**  How can we best support the study participant? |
| --- | --- | --- |
| Body image issues | E.g. Lowered self-esteem, increased focus on body image etc. | **Beyond Body Image** <https://www.beyondbodyimage.com/resources/>  This website contains information about a wide range of resources supporting positive body image including books, website and podcasts.  **Be Body Positive** <https://bebodypositive.org.uk/>  A UK based resource containing free modules for young people looking to build a positive body image  **10 Steps to Positive Body Image** <https://www.nationaleatingdisorders.org/learn/general-information/ten-steps>  Some useful tips about building a positive body image.  **The Body Positive** <https://thebodypositive.org/>  A US based non-profit organisation ‘Imaging a world where people in all bodies are able to reclaim their health, beauty and confidence to live full, happy lives’ |
| Body image issues | E.g. altered / lowered mood | **Mind**  <https://www.mind.org.uk/>  ‘We support minds – offering help whenever you might need it through our information, advice and local services’    **Samaritans** <https://www.samaritans.org/>  Call 116 123 (calls are free and don’t appear in call records) or email [jo@samaritans.org](mailto:jo@samaritans.org). There’s also a self-help app and online chat.  Provides a non-judgemental space to talk and be listened to.  *NB it may be appropriate to signpost to their GP. They may already have mental health support systems in place.* |
| Frustration, dissatisfaction, anger | E.g. would like to make their concerns known | **How to raise concerns / make a complaint**  **Mind**  <https://www.mind.org.uk/information-support/legal-rights/complaining-about-health-and-social-care/overview/>  Information about how to provide feedback and / or to make a complaint about healthcare. Information is included for all sectors i.e. private as well as healthcare. |

**Signposting Log**

| **Date resources sent** | **Researcher name** | **Summary of topic raised by study participant** | **Signposting resources provided** |
| --- | --- | --- | --- |
|  |  |  |  |
|  |  |  |  |
|  |  |  |  |
|  |  |  |  |

Dear [name used in contact email],

Thank you for contacting us, we are sorry if participating in the survey has brought up some unsettling feelings. As I am not a trained professional in these matters, I am unable to provide support personally, but I can signpost to other services that may be of use.

In your email you indicated struggling with [XXXXXX], therefore I have the following suggestion of people and organisations who may be able to help:

1. [option 1]
2. [option 2]
3. Etc

I hope that one of these will be able to provide the support you need.

Best wishes

[Researchers name]
